# Recent Developments in Nanoparticle Formulations for Resveratrol Encapsulation as an Anticancer Agent

**DOI:** 10.3390/ph17010126

**Published:** 2024-01-18

**Authors:** Muhammad Ali, Viviana Benfante, Domenico Di Raimondo, Giuseppe Salvaggio, Antonino Tuttolomondo, Albert Comelli

**Affiliations:** 1 Ri.MED Foundation, Via Bandiera 11, 90133 Palermo, Italy; amuhammad@fondazionerimed.com; 2 Department of Health Promotion, Mother and Child Care, Internal Medicine and Medical Specialties, Molecular and Clinical Medicine, University of Palermo, 90127 Palermo, Italy; domenico.diraimondo@unipa.it (D.D.R.); bruno.tuttolomondo@unipa.it (A.T.); 3 Department of Biomedicine, Neuroscience and Advanced Diagnostics, University of Palermo, 90127 Palermo, Italy; p.salvaggio@libero.it; 4 National Biodiversity Future Center (NBFC), 90133 Palermo, Italy

**Keywords:** anticancer properties, bioavailability, nanoparticles, polyphenolic compound, resveratrol encapsulation

## Abstract

Resveratrol is a polyphenolic compound that has gained considerable attention in the past decade due to its multifaceted therapeutic potential, including anti-inflammatory and anticancer properties. However, its anticancer efficacy is impeded by low water solubility, dose-limiting toxicity, low bioavailability, and rapid hepatic metabolism. To overcome these hurdles, various nanoparticles such as organic and inorganic nanoparticles, liposomes, polymeric nanoparticles, dendrimers, solid lipid nanoparticles, gold nanoparticles, zinc oxide nanoparticles, zeolitic imidazolate frameworks, carbon nanotubes, bioactive glass nanoparticles, and mesoporous nanoparticles were employed to deliver resveratrol, enhancing its water solubility, bioavailability, and efficacy against various types of cancer. Resveratrol-loaded nanoparticle or resveratrol-conjugated nanoparticle administration exhibits excellent anticancer potency compared to free resveratrol. This review highlights the latest developments in nanoparticle-based delivery systems for resveratrol, focusing on the potential to overcome limitations associated with the compound’s bioavailability and therapeutic effectiveness.

## 1. Introduction

Cancer represents a significant global health challenge, standing as the second most common and prevalent cause of mortality. According to the World Health Organization (WHO), 10 million people died in 2020, a figure projected to increase by 70% in the next two decades [1,2]. The estimated worldwide economic cost of cancer is USD 25.2 trillion for 2020 to 2050 [3]. Conventional treatments include chemotherapy, immunotherapy, radiotherapy, and surgery [4,5]. However, modalities such as chemotherapy and radiotherapy encounter challenges in the form of radioresistance and chemoresistance. To overcome these limitations, exploring innovative therapeutic molecules and advancing drug delivery systems is imperative to effectively treat various cancer forms [6,7].

Plant-derived sources present an alternative reservoir of bioactive compounds with potential applications of therapeutic or prophylactic agents against various diseases [8,9,10,11,12,13,14,15]. To date, 350,000 vascular plant species are known, and new species are discovered yearly [16]. It is still a broad and understudied area of research with many prospects for new therapeutic development. However, active substances can be extracted and serve as valuable resources for medicinal applications and as building blocks for synthetic and semi-synthetic substances [17]. Among the diverse array of phytochemicals, encompassing terpenes, alkaloids, essential oils, flavonoids, gums, and a range of primary and secondary metabolic components, discernible medicinal effects have been identified [18,19]. A notable statistic underscores the significance of natural origins, indicating that 51% of the 1211 newly approved small-molecule drugs worldwide between 1981 and 2014 were derived from natural products [20]. Polyphenols have been known to have various preventive effects on different conditions such as diabetes, cardiovascular disease, neurodegenerative disorder, and obesity [21,22,23,24,25,26,27,28,29], and have been extensively studied to determine their anticancer potential and incorporate them into cancer treatment modalities like chemotherapy and targeted therapy [30,31,32,33].

Resveratrol (RSV), a stilbenoid polyphenolic compound, has emerged as a promising anticancer agent. However, its therapeutic potential is hindered due to its pharmacokinetic properties, such as chemical instability (due to oxidation and photosensitivity), low water solubility, low bioavailability, rapid metabolism, and elimination [34,35,36,37]. Many attempts have been made to overcome these hurdles using nanoparticles. The encapsulation of resveratrol in nanoparticles increases its absorption, bioavailability, and sustained release [38,39,40,41,42,43,44]. Cutting-edge nanoparticle technology has revolutionized engineers’ and scientists’ approaches to various fields of study. Nanoparticles are advancing the development of novel drug delivery systems, material engineering, and diagnostic sciences [45,46,47,48]. This review aims to highlight the therapeutic effect of RSV using nanoparticle delivery systems and the potential for cancer therapy against various types of cancer, e.g., brain, prostate, skin, breast, lung, colon, liver, pancreas, ovarian, and gastric cancers, by evaluating in vitro and in vivo studies through an overview of recent progress.

## 2. Resveratrol

Resveratrol (RSV) (3,5,4′-trihydroxystilbene) is a well-known naturally occurring polyphenolic compound present in various types of plants such as legumes, blueberries, cranberries, grapes, eucalyptus, and various grasses [49,50]. RSV is a secondary metabolite in different plant families, such as Gnetaceae, Dipterocarpaceae, Leguminosae, and Cyperaceae. Moreover, plants produce RSV in response to pathogen attacks, UV radiation, damage, stress, and exposure to ozone [51]. RSV can be modified into various structures, e.g., pterostilbene, 4,4′-hydroxy-trans-stilbene, monoalkoxy, dialkoxy derivatives, and trans 3,4′,5-trimethoxystilbene [52]. RSV has been confirmed to have many health benefits, such as antiviral, antioxidant, anti-inflammatory, neurological, and heart-disease-prevention properties [53,54,55,56]. RSV also enhances the antiviral activity of various drugs, such as zidovudine, zalcitabine, and didanosine [57,58,59]. The anticancer properties of RSV against multiple malignancies were first described by Jang et al. in 1997 [60].

### 2.1. The Structure and Physical Properties of Resveratrol

**Figure 1:**
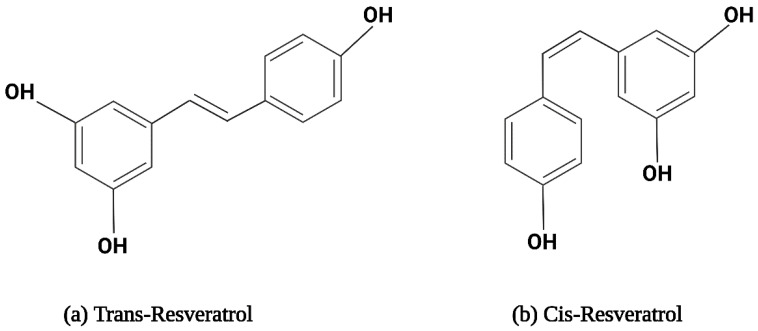
The structural formula for resveratrol isomers.

Resveratrol is a hydrophobic compound characterized by a molecular weight of 228.25 g/mol and a melting point of 254 °C [61]. The solubility of RSV is 30 µg/mL in water. RSV is soluble in polar compounds, especially dimethyl sulfoxide (DMSO) and ethanol [61,62]. It exists in two isoforms, i.e., trans-RSV and cis-RSV, depicted in Figure 1. The trans-isomer is more stable and predominant, with more therapeutic properties [63]. Cytotoxicity studies on pancreatic cancer, breast cancer, small-cell lung carcinoma, colon cancer, and prostate cancer cell lines revealed that trans-resveratrol possesses slightly more potent cytotoxic properties than the cis-isomer, attributed to its better bioavailability and biodistribution [64,65,66]. Upon exposure to sunlight or UV radiation at 254 nm or 366 nm, trans-RSV converts into cis-RSV and vice versa. Trans-RSV is more thermo- and photo-stable than cis-RSV. Trans-RSV remains stable in neutral aqueous buffers for 42 h and 28 days at acidic pH; however, cis-RSV remains stable at neutral pH [67,68]. Additionally, RSV becomes unstable when exposed to high humidity and prolonged exposure to light [69]. Additionally, there is evidence that RSV oxidizes into quinines and semiquinones, which cause cell damage [70].

### 2.2. Metabolism of Resveratrol

RSV has a half-life of 8 to 14 min in plasma after oral treatment, and its plasma concentrations are often low, sometimes not detectable at all [71]. Upon oral administration, RSV is absorbed by enterocytes, which undergo sulfate conjugation and glucuronidation in the liver and intestine, leading to the formation of trans-resveratrol-3-sulfate and trans-resveratrol-3-O-glucuronide metabolites [36]. Meanwhile, a small quantity of free RSV remains in the blood circulation after being absorbed by plasma proteins like albumin, blood cells, and lipoproteins [72]. Primarily, RSV is administered orally; different levels of free RSV can be detected in urine, ranging from negligible quantities to 17%. The sulfated form of cis-RSV-4′-sulfate is more prevalent than the glucuronidated form. In addition, several studies have found that a minor amount of RSV metabolites are excreted in human feces [73,74].

### 2.3. Mechanism of Action of Resveratrol against Cancer

**Figure 2:**
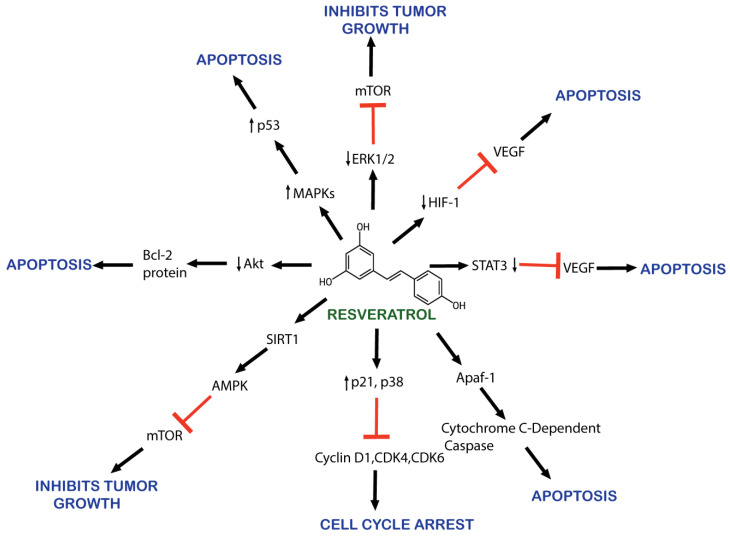
Schematic representation of resveratrol’s anticancer effects.

RSV is known for its anticancerous properties, mediating apoptosis, cell growth, metastasis, and angiogenesis, as illustrated in Figure 2 [75,76,77,78,79,80,81,82]. Its mechanism involves reducing angiogenesis and increasing apoptosis through the inhibition of vascular endothelial growth factor (VEGF) expression by downregulating hypoxia-inducible factor 1 (HIF-1) [76,77]. RSV promotes apoptosis by arresting the cell cycle at G_0_/G_1_ by upregulating the expression of cyclin-dependent kinase (CDK) inhibitors p21 and p27. It also upregulates the expression of cyclin D1, CDK 4, and CDK6 [83].

RSV causes the apoptosis of cancerous cells by downregulating HER2/neu expression [84]. RSV also inhibits cancer-promoting molecular pathways, such as nuclear factor kappa B (NF-kB), PI3K/AKT/mTOR, and STAT3 [85]. AKT serine/threonine kinase is an oncogene protein involved in cell survival, apoptosis, proliferation, and growth. It is involved in the phosphoinositide 3-kinase (PI3K)/AKT signaling pathway activated by inflammation, DNA damage, and growth factors [86]. AKT overexpression has been observed in various cancers [87,88]. RSV also inhibits NF-kB-regulated genes such as VEGF, B cell lymphoma protein-2 (Bcl-2), B-cell lymphoma-extra-large (Bcl-xL), and matrix metalloproteinase (MMP). As NF-kB activates, it alters caspase activity and increases antiapoptotic gene expression, promoting cell proliferation and protecting cells from apoptosis [89].

Moreover, RSV also activates the p53 kinase mediated by MAPKs (mitogen-activated protein kinases) [90]. RSV induces the activation of Apaf-1, which involves activating cytochrome C-dependent caspase and triggers a cascade of apoptosis events [91]. RSV inhibits the activity of the cyclooxygenase enzyme (COX) which converts arachidonic acid to prostaglandin, an inflammatory factor that induces tumor cell proliferation [92,93]. RSV also activates the SIRT1 enzyme, which deacetylates histone and non-histone proteins. SIRT1 regulates inflammation, cell cycle defects, and metabolic control [94,95]. In vitro research demonstrates RSV’s efficacy at reducing cell proliferation and promoting apoptosis by downregulating molecular targets, including p-Akt, cyclin D1, the mammalian target of rapamycin, and androgen receptor (AR) protein [96].

Despite its therapeutic potential, RSV has low gastrointestinal (GI) absorption because of its low water solubility and quick metabolism, and the degradation of RSV by oxidative enzymes [97]. Moreover, cancer cells can develop resistance to chemotherapeutic drugs due to mutations known as multidrug resistance (MDR). Other carcinogenesis processes lead to MDR, including pathways leading to apoptosis, DNA damage response, downstream signaling pathways, changes in drug efflux attributable to modifications in proteins involved in drug transfer from the cell membrane, changes in enzymes involved in drug processing and metabolism, changes in the composition of the cell membrane, cancer stem cells (CSCs), epithelial–mesenchymal transition, and changes in the tumor environment [98].

A potential solution to these problems involves the development of nanoparticles capable of carrying resveratrol, ensuring targeted delivery without inducing toxicity [97].

Critical considerations in nanoparticle design, crucial for an effective drug delivery system, include the following:

1. Nanoparticles should enhance free molecules’ specificity, efficacy, therapeutic index, and tolerability [99].
2. Nanoparticles must be non-toxic, non-immunogenic, non-thrombogenic, and biodegradable [100].
3. They should also protect and maintain active compounds’ structural integrity and enhance their bioavailability [40,101,102].

## 3. Application of Nanoparticles to Improve the Therapeutic Potential of Resveratrol for Cancer

In nanotechnology, a particle is often categorized according to its physical diameter: ultrafine particles typically have a physical diameter between 1 and 100 nm in at least one dimension [40]. Drugs can be conjugated to nanoparticle surfaces through covalent or ionic bonds, structural absorption, or encapsulation inside nanoparticle cores [103]. These nanoparticle-based formulations can increase absorption, bioavailability, and chemical integrity, enhance permeability and retention effect (EPR) across the biological membrane, and ensure the optimal dosage of drugs reaches the cancer target cells [104,105]. Biodistribution studies have demonstrated that RSV-loaded nanoparticles or RSV-conjugated nanoparticles have a much longer circulation time than free RSV [106,107,108,109]. Nanoparticles protect RSV from rapid metabolism and elimination, resulting in sustained blood levels. According to pharmacokinetic studies, nanoencapsulated RSV has an extended half-life [110]. Pharmacokinetic studies have also revealed that RSV bioavailability is increased when delivered in nanoformulations. Nanoparticles improve RSV absorption in the gastrointestinal tract and protect first-pass metabolism in the liver [111,112,113]. Biodistribution studies revealed that nanoparticles enhanced RSV accumulation in tumor tissues compared to free RSV due to enhanced permeability and retention (EPR) in tumor vasculature. Nanoformulations provide controlled and sustained RSV release. The controlled release of RSV contributes to prolonged therapeutic effects and decreases the need for frequent dosing [114,115].

Various strategies, e.g., triggered drug release and stimuli-responsive approaches, have been used in RSV nanoformulation to release RSV in response to specific internal or external triggers. These strategies aim to increase therapeutic efficacy and precise control, and minimize the side effects of drug release. The common stimuli-responsive approaches are pH-responsive release, enzyme-responsive release, temperature-responsive release, redox-responsive release, light-responsive release, magnetic-responsive release, and ultrasound-responsive release (Table 1) [116].

In pH-sensitive nanoparticles, RSV is released in response to acidic pH conditions in tumor microenvironments. This pH-triggered release enhances drug delivery to cancer cells while minimizing release in normal tissues [117]. Thermosensitive nanoparticles loaded with RSV release the drug in response to local temperature changes. Hyperthermia treatment can trigger drug release [118]. The enzyme-triggered release of RSV can be achieved by conjugating enzyme-cleavable compounds in the nanoparticle structure. The presence of disease-specific enzymes, such as matrix metalloproteinases in tumors, releases RSV [119]. Redox-responsive nanoparticles release RSV in response to elevated reactive oxygen species (ROS) at the tumor site. Incorporating photo-responsive materials into resveratrol nanoparticles allows light-induced drug release [120]. Resveratrol-loaded magnetic nanoparticles can be guided to specific target sites using external magnets. Magnetic fields induce drug release at the desired location [121]. Ultrasound-responsive nanoparticles loaded with resveratrol can be triggered to release the drug at the target site using focused ultrasound waves, providing spatial and temporal control. These stimuli-activated approaches offer precise control over drug-release kinetics, improving resveratrol’s therapeutic index and minimizing systemic side effects. The trigger mechanism’s choice depends on the target tissue’s specific characteristics and the desired therapeutic outcome [122].

**Figure 3:**
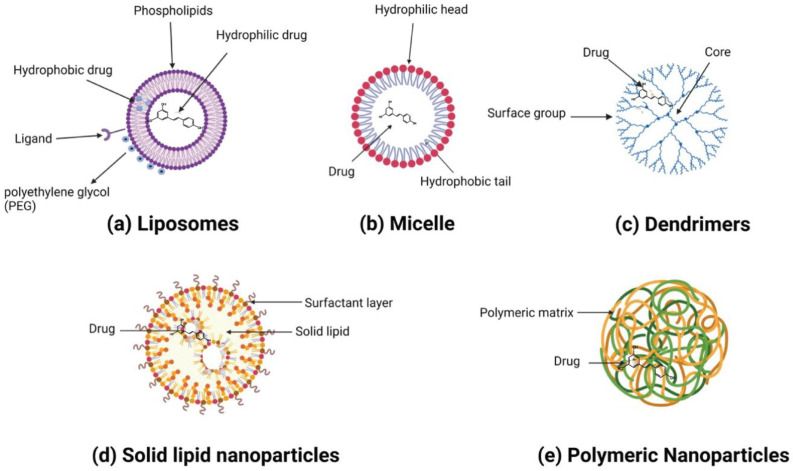
A variety of resveratrol-loaded organic nanoparticles.

**Figure 4:**
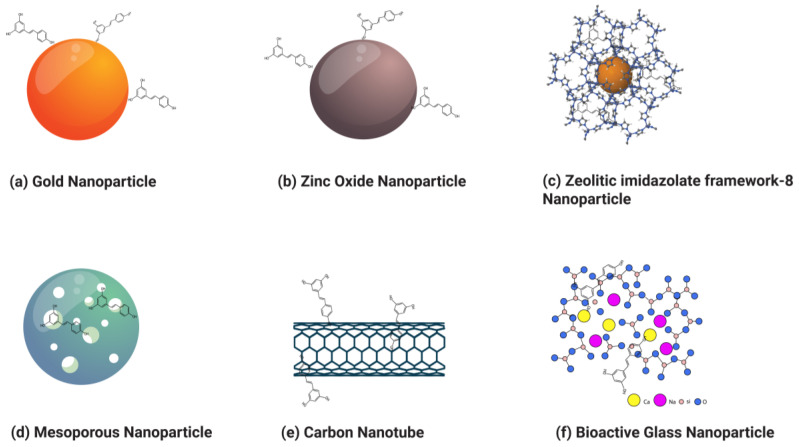
Various types of resveratrol-loaded/conjugated inorganic nanoparticles.

Nanoparticles can broadly be categorized into organic and inorganic types, with recent extensive studies focusing on organic particles. Specifically, liposomes, polymersomes, polymer constructions, and micelles are used for imaging and drug and gene delivery methods (Table 2). Although inorganic nanoparticles exhibit highly material- and size-dependent physicochemical characteristics, incomparable with conventional lipid- or polymer-based NPs, they have also attracted researchers’ interest in recent years (Table 3) [123]. Various nanoformulations, including liposomes, metallic nanoparticles, solid lipid nanoparticles, micelles, polymeric nanoparticles, and inorganic nanoparticles, are illustrated in Figure 3 and Figure 4.

Several attempts have been made to develop nanotechnology-based strategies and increase bioavailability and effectiveness in various cancer models [40,41,42,43,44,124]. In an initial attempt at encapsulating RSV, chitosan NPs were used. The study conducted by Yao et al. showed that sustained release of RSV, with resveratrol-loaded nanoparticles at lower concentrations, caused an increased percentage of cell death compared to an equivalent dose of free resveratrol [125].

### 3.1. Organic Nanoparticles

#### 3.1.1. Liposomes

Liposomes are spherical vesicles consisting of a phospholipid bilayer and an aqueous core. Hydrophobic drugs can be encapsulated in the phospholipid bilayer, while hydrophilic drugs can be loaded into an aqueous core. The liposome structure can be modified to obtain the desired therapeutic effect. Small molecules such as antibodies and ligands can be attached to liposomes to target specified cells. Drug release is possible under specific pH, enzyme, and ultrasound conditions. Liposomes protect drugs from photodegradation, e.g., when exposed to UV light. Similarly, trans-resveratrol (70%) remains intact in liposomes for 16 min compared to free RSV [126]. The methodology used to prepare liposomes depends upon the desired characteristics of the liposomes, such as half-life, size, drug properties, solvent type, cost, and liposome components [127]. Liposomes are synthesized using thin-film hydration. In the thin-film hydration technique, a lipid is dissolved in an organic compound, evaporation occurs, and the obtained film is dispersed. Some other methods include reverse-phase evaporation, dehydration–rehydration, solvent injection, and microfluidic-based techniques for high encapsulation [128]. There is another type of liposome called magnetoliposomes which have a magnetic core in the lipid bilayer. Magnetoliposomes can be excited by a magnetic field, creating hyperthermia within cancerous cells [129].

RSV and quercetin co-encapsulated in liposomes have been used to study cancer mouse models’ anti-inflammatory and antioxidative responses. Liposomes increase antioxidant activity and decrease leukocyte infiltration, edema, and tissue damage [130]. Narayanan and colleagues reported liposome-encapsulated RSV in B6C3F1/J mice; nanocarriers were found to reduce the incidence of prostate carcinoma compared to free RSV [96]. Jhaveria et al. constructed RSV-loaded liposomes and investigated their antiproliferative activity against U-87 MG cells. Hence, it was concluded that these nanoparticles had excellent anticancer activity compared to free RSV [131]. In another study, RSV-loaded liposomes showed excellent anticancer efficacy in xenografted resistant A549/cDDP nude mouse models [132]. Meng et al. synthesized liposomes coloaded with paclitaxel and RSV. The liposomes’ average size was 50 nm, and the encapsulation efficiency was above 50%. The study’s key findings were that liposomes improved tumor retention and bioavailability in mice with drug-resistant tumors and induced cytotoxicity in drug-resistant MCF-7/ADR tumor cells [133].

Nanoparticle surfaces can be conjugated with various targeting ligands, e.g., peptides, antibodies, and aptamers. Thus, the nanoparticles reach the tumor site, target the tumor cells, and release the drug for enhanced efficacy [134]. The outer surfaces of nanoparticles can be conjugated with a polyethylene glycol (PEG) called PEGylation. These PEGylated nanoparticles remain in the blood circulation for a long time and protect biotransformation reactions [135]. In one study, a PEGylated RSV-phospholipid bilayer enveloping a casein micelle structure was constructed. This nanocarrier showed high cytotoxicity in the MCF-7 cancer cell line compared to free RSV, showing effective anticancer activity in tumor-bearing mice [136]. In another study, PEGylated liposomes were used as a delivery system to examine the therapeutic synergy between RSV and 5-fluorouracil. The nanoformulation had a GI_50_ comparable to free 5-fluorouracil when tested in vitro on the NT8e cell line [42].

Cancer cells express transferrin receptors (TfRs) more widely than normal cells. As a result, they require an increased amount of iron. A serum glycoprotein called transferrin (Tf) participates in iron transfer into cells by interacting with a receptor specific to Tf (TfR). TfR is, therefore, suitable for the direct selection of drug delivery to cancer cells because of its high levels of expression in these cells [137]. In comparison to RSV-PEGylated liposomes, Tf-targeted resveratrol-loaded liposomes (Tf-resveratrol-L) enhanced cytotoxicity, increased the apoptosis rate in glioblastoma (GBM) cells, and decreased tumor growth in mice [131].

Poonia et al. conjugated folic acid to the RSV nanostructure. The folate-targeted nanostructure showed high cytotoxicity on the MCF-7 cell line compared to the unmodified nanostructure. These nanostructures were delivered intravenously to rats, revealing that folate-targeted nanocarrier bioavailability was nine times higher than free RSV [138]. Similarly, an RSV-loaded mixed-micelle nanocarrier was constructed in the same study, and PEG and folic acid were combined on the nanocarrier surface. The nanocarrier’s diameter was 20 nm, and it was administered to rats. The folic-acid-conjugated nanostructure showed a plasma RSV level four times higher than the free RSV [138].

**Figure 5:**
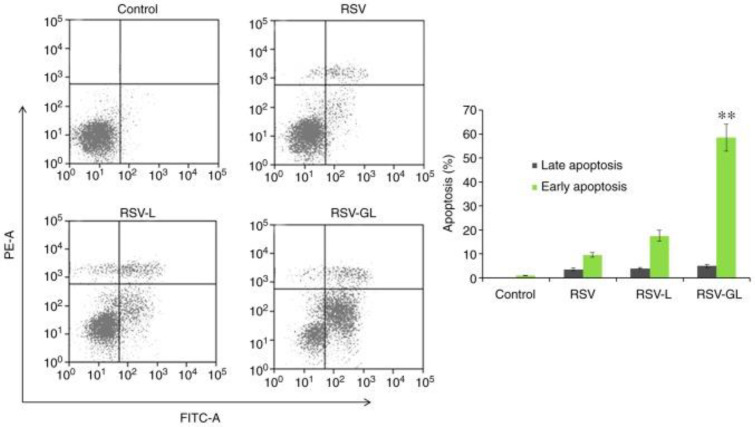
The therapeutic efficacy of RSV, RSV-L, and RSV-GL in squamous cell carcinoma (SCC7) cell line. Annexin V-FITC/PI staining was used to study the apoptosis of cancer cells. Reprinted from Ref. [141], Copyright 2019, *International Journal of Molecular Medicine*. ** *p* < 0.01 vs. RSV-L. This work is licensed under Attribution-NonCommercial-No Derivatives 4.0 International (CC BY-NC-ND 4.0).

**Figure 6:**
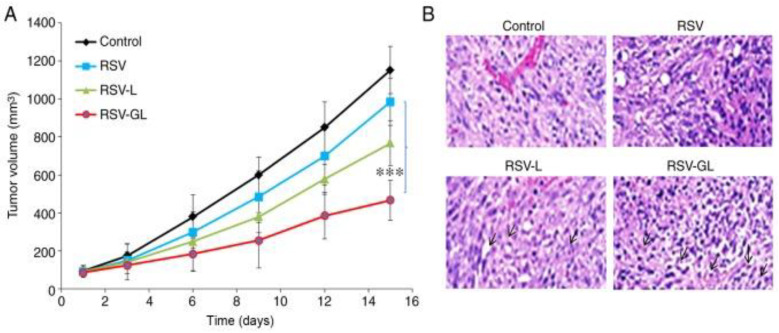
In vivo analysis of RSV, RSV-L, and RSV-GL in SSC-bearing xenograft model. (**A**) Tumor volume; (**B**) hematoxylin and eosin histology staining analysis. *** *p* < 0.0001 vs. RSV. Reprinted from Ref. [141]. Copyright 2019, *International Journal of Molecular Medicine*. This work is licensed under Attribution-NonCommercial-No Derivatives 4.0 International (CC BY-NC-ND 4.0).

In another study, resveratrol-loaded liposomes modified with folate (FA-RSV-liposomes) were synthesized to analyze anticancer activity against the human osteosarcoma cell 143B. According to the study, FA-RSV-liposomes promoted apoptosis and inhibited tumor cell proliferation. Folate-modified liposomes showed significant anticancer activity compared to free RSV [139]. Wang and co-workers synthesized PEGylated-RSV liposomes combined with and without glycine and determined their anticancer efficacy against HeLa and MCF-7 cells. The results revealed a higher drug-entrapment efficiency for glycine-containing liposomes than non-glycine-containing liposomes [140]. In one study, Zheng et al. constructed a liposome conjugated with PEG and dodecapeptide (GE11) to increase RSV’s therapeutic effect against head and neck cancer in vitro and in vivo. RSV-loaded GE11-conjugated liposomes (RSV-GL) showed a high entrapment efficiency of >95%. The epidermal growth factor receptor (EGFR) was overexpressed in squamous cell carcinoma HN cells, which internalize GE11-conjugated liposomes. RSV-GL showed increased cytotoxicity compared to the non-targeted nanoparticles (Figure 5 and Figure 6) [141].

#### 3.1.2. Polymeric Nanoparticles

Polymeric nanoparticles consist of a polymer matrix, such as polysaccharides, poly(acrylic acid), poly(lactic-co-glycolic acid) (PCL), poly(lactic acid) (PLA), poly(ε-caprolactone) (PCL), poly(methacrylic acid), poly L-lysine (PLL), poly amidoamine (PAMAM), and polypropylene imine (PPI), zein, gelatin, albumin, and silk [142]. Polymer-based nanoparticles can be synthesized by various methods such as solvent diffusion, solvent evaporation, ionic gelation, self-assembly, polymer electrostatic interaction, desolvation, and emulsion [143]. The drug is conjugated within a polymeric matrix structure. A nanoparticle protects the drug from degradation, provides sustained drug release, and enhances its effectiveness [144]. The structure of polymeric nanoparticles can be modified to target tumor cells [145]. RSV incorporation into improved polymeric NPs offers many advantages, such as controlled drug release and defense against light-exposure degradation. Neves et al. designed solid lipid NPs to enhance RSV’s oral bioavailability [41]. Sanna et al. synthesized RSV-loaded polymeric nanoparticles by using a blend of two biocompatible polymers: (i) poly(epsilon-caprolactone) (PCL) and (ii) poly(D, L-lactic-co-glycolic acid)-poly(ethylene glycol) (PLGA-PEG-COOH) conjugate. Prostate cancer was treated with these polymers, and only 55% of the RSV was released within 7 h. At pH 6.5 and 7.4, NPs released 55% of their total RSV in simulated gastrointestinal fluids within the first two hours. The remaining 74% was released within five hours at pH 7.4. Confocal microscopy observations showed that PCa cell lines effectively absorb NPs [124]. 

Studies on nanoparticle formulations, biodistribution, and in vivo pharmacokinetics can potentially shed light on their safety. The syntheses of mixed micelles P127/TPGS [146] and piperidine-loaded mixed micelles both showed the benefits of RSV’s gradual release, avoiding adverse effects [147]. Biodistribution studies have proven that organ buildup formulations indirectly reveal off-target side effects. For example, it has been demonstrated that folic-acid-conjugated P127/TPGS mixed micelles reduce accumulation in various organs [146], potentially reducing the associated adverse effects of RSV. Additionally, it was shown that glyceryl behenate-based SLN [106], TPGS-coated nanoparticles, and PLGA: TPGS blended nanoparticles conjugated with folic acid enhance cancerous cell targeting [148].

Various studies have investigated the anticancer properties of newly created RSV-loaded polyethylene glycol-polylactic acid (PEG-PLA; MW 5000-5000) polymer nanoparticles. The main findings included a significant drop in cell quantity of CT26 colon cancer cells to 5.6% and colony-forming capacity to 6.3% after 72 h of treatment with 40 and 20 M of RSV nanoformulation, as well as an increase in ^18^F-FDG absorption and a decrease in ROS levels [149,150]. Jung and their co-workers used 18F-fluorodeoxyglucose (18F FDG) as a biomarker for monitoring RSV-loaded NPs in colon cancer cells bearing BALB/c nude mice. In another study, Zhao et al. observed that RSV encapsulated in PLGA NPs reduced tumor growth in MCF-7 and MDA-MB cancer-bearing mice. They also observed that the RSV nanocarrier showed significant anticancer efficacy in CT26 colon cancer cells [151].

**Figure 7:**
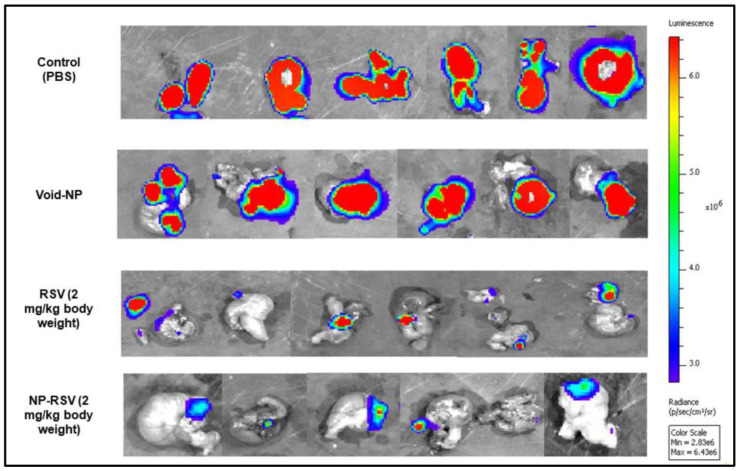
Ex vivo IVIS image analysis of the orthotopic COLO205-luc-bearing animal model. High-signal-intensity (red color) areas indicate increased cancer cell viability. Blue-color areas indicate the lowest viability. Reprinted from Ref. [152]. Copyright 2020, MDPI. This article is an open access article distributed under the terms and conditions of the Creative Commons Attribution (CC BY 4.0) license.

In one study, Sudah et al. synthesized RSV encapsulated in poly glycol–lactic acid–polyethylene glycol (PLGA-PEG) NPs conjugated with chitosan and injected in orthotopic mouse models with colon cancer (COLO205-luc). The results showed that NP-RSV decreased tumor growth by reducing angiogenesis in mouse models (Figure 7) [152].

Aldawsari et al. developed chitosan (CS)-coated PLGA nanoparticles for RSV (RSV-CS-PLGA NPs). Their anticancer efficacy was evaluated in H1299 lung cancer cell line. The CS coating provided stability to RSV-loaded PLGA nanoparticles. Comparing CS-coated PLGA NPs with PLGA NPs and free RSV, CS-coated PLGA NPs showed better drug solubility, stability, sustained release, and therapeutic potential [153].

In another study, Zhang et al. constructed nanoparticles by loading RSV in poly(ε-caprolactone)–poly(ethylene glycol) (PCL-PEG) nanoparticles with an erythrocyte membrane (RSV-NPs-RBCm) and injected them in an HT29 xenograft mouse model. The results revealed that RSV-NPs-RBCm have higher bioavailability and anti-tumor activity than free RSV. Moreover, the RSV-NPs-RBCm were conjugated with iRGD, which enhanced tumor tissue penetration [154]. The iRGD peptide is a short sequence of amino acids: Arg-Gly-Asp. This peptide was discovered by phage display and is a tumor-targeting peptide. Its ability to penetrate tumor cells can enhance tissue penetration to enhance therapeutic efficacy and image sensitivity [155,156]. Similarly, Geng et al. synthesized RSV-loaded human serum albumin (HSA) nanoparticles conjugated with RGD via PEG and analyzed their anticancer effect on PANC-1 cells and Balb/c nude mice. The results revealed that RSV-loaded HSA-RGD nanoparticles showed the highest cellular uptake of 47.3% compared to RSV-loaded HSA nanoparticles. RSV-loaded HAS-RGD nanoparticles remained in the blood circulation for a long time and were retained in tumor tissue three and eight times more effectively than RSV-loaded HAS and free RSV [157].

In another study, Long et al. synthesized RGD-conjugated RSV-containing HAS nanoparticles (RSV-HSA-RGD NPs) and evaluated their anticancer efficacy against SKOV3 ovarian cancer cells in a mouse model. The RSV-HSA-RGD NPs showed better anticancer activity than HAS-RVT NPs and free RSV [158].

**Figure 8:**
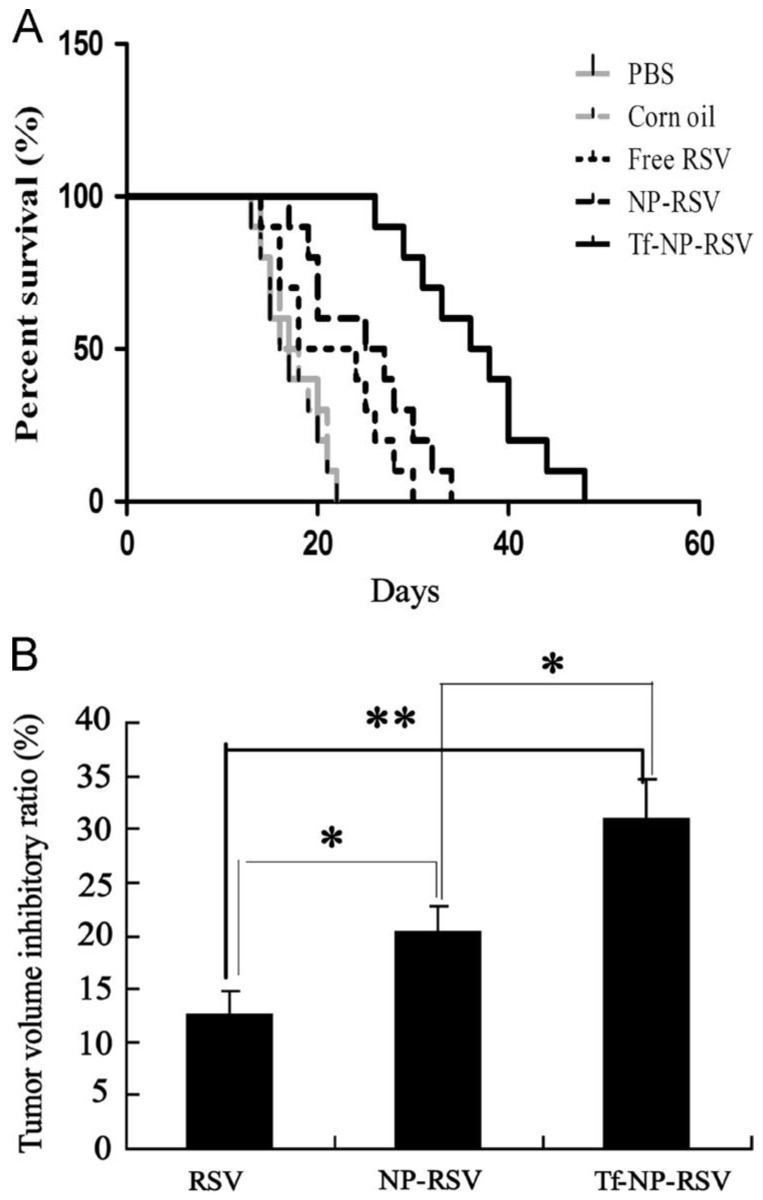
The effect of RSV conjugates on survival and tumor volume in C6 glioma-bearing rats. (**A**) In vivo effects of RSV and RSV-polymer conjugates after injection intraperitoneally at a dose of 15 mg RSV-equiv./kg on the survival of brain-tumor-bearing rats. (**B**) Inhibitory ratios of tumor volume in brain-glioma-bearing rats after treatment with RSV solution and RSV-polymer conjugates. * *p* < 0.05, ** *p* < 0.01. Reprinted with permission from [159]. Copyright 2013, Elsevier.

Guo et al. evaluated the anticancer efficacy of resveratrol-loaded transferrin-modified polyethylene glycol-polylactic acid nanoparticles (Tf-PEG-PLA-RSV) in C6 glioma-bearing rats. They observed that nanoparticles conjugated with RSV showed good anticancer activity compared to free RSV, decreasing tumor volume and accumulation in tumor cells (Figure 8) [159].

Hussain et al. evaluated the anticancer efficacy of RSV-loaded soluplus polymeric nanoparticles (PNPs) against C6 glioma cell lines. Soluplus (polyvinyl caprolactam-polyvinyl acetate-poly-ethylene glycol) is a copolymer with a hydrophobic core that delivers lipophilic compounds. Soluplus and D-α-tocopheryl polyethylene glycol 1000 succinate (TPGS1000) nanoparticles increase blood–brain barrier permeation. The study revealed that RSV-loaded PNPs enhanced bioavailability and increased anti-glioma activity more than free RSV [160].

In another study, Karthikeyan et al. developed resveratrol-loaded gelatin nanoparticles which showed excellent anticancer efficacy in the NCL-H460 cell line compared to free RSV. They also observed that the nanocarrier bioavailability was two times greater than free RSV in Swiss albino mice [161]. A cross-linked zein nanoparticle was constructed by Elzoghby et al. for the co-delivery of RSV and exemestane. It was revealed that the nanoformulation of both drugs decreased tumor volume by 2.4 times more than free drugs [162]. According to Lu et al., the pre-incubation of resveratrol-loaded polymeric micelles for 12 h protected PC12 cells from amyloid peptide (Abeta)-induced damage by reducing caspase-3 activity and intracellular oxidative stress, affecting apoptosis without long-term cytotoxicity [163].

Dendrimers exhibit a homogeneous, well-defined, and monodisperse structure composed of tree-like branches or dendrons, with a central core surrounded by surface groups [164]. Dendrimers have a predictable size, weight, structure, and shape. Dendrimers can be modified to enhance the bioavailability, stability, and solubility of drugs and target tumor cells [165]. Repeated monomers are used to create dendrimers. They can be synthesized from different compounds, such as poly L-lysine (PLL), poly amidoamine (PAMAM), and polypropylene imine (PPI) [166]. Dendrimers can be synthesized using a convergent or divergent method [167].

Dendrimers can transport RSV through non-covalent and covalent interactions. In noncovalent interactions, RSV can be wrapped within the internal structure of the dendrimer to protect it from metabolization and help it reach the target site by increasing its bioavailability. In covalent interactions, RSV can be covalently lined with dendritic polymers such as amines, carbamates, and esters to control drug release [168]. Scientists grafted RSV-loaded nanoparticles with PAMAM modified by lactose acid (LA) (LA-PAMAM-RSV) to evaluate the anticancer efficacy in vitro. LA-PAMAM-RSV nanoparticles showed sustained drug release, biocompatibility, and anticancer activity compared to free RSV [169].

**Figure 9:**
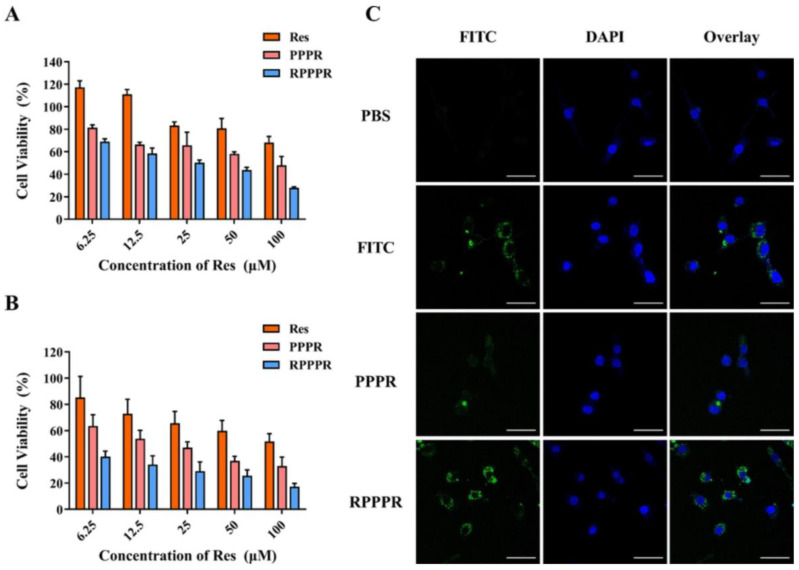
The effect of RSV, PPPR, and RPPPR on L929 cells (**A**) and CT26 cells (**B**). (**C**) In vitro targeting and cellular uptake of nano-prodrugs. Confocal laser scanning microscopy (CLSM) images of CT26 cells after 24 h incubation with PBS, free FITC, FITC-labeled PPPR, and FITC-labeled RPPPR, respectively. Reprinted with permission from [170]. Copyright 2023, Elsevier.

Gu et al. used PAMAM dendrimer nanoparticles to deliver RSV in vitro and in vivo. The PAMAM dendrimer was modified with PEG, RGDyC, and 4-Hydroxy-phenylboronic acid (PBA). The in vitro cytotoxic properties of RGDyC-PEG-PAMAM-PBA-RSV (RPPPR) and PEG-PAMAM-PBA-RSV (PPPR) were evaluated in murine CT26 and L929 cell lines, which revealed significant cytotoxic properties compared to free RSV (Figure 9). Meanwhile, in vivo findings showed apoptosis and the inhibition of tumor cell growth without toxicity to the vital organs [170]. In another study, RSV was loaded into a silica and PAMAM G4 hybrid matrix. RSV-loaded NPs inhibited INOS, with an IC_50_ of 249.74 µM against estrogen-positive and -negative breast cancer cells [171].

#### 3.1.3. Solid Lipid Nanoparticles

Solid lipid nanoparticles (SLNs) are spherical vesicles with 50–1000 nm diameters, consisting of a surfactant layer and a lipid core [172]. SLNs are synthesized by replacing liquid lipids with lipids in a water/oil emulsion [173]. Hydrophobic drugs can be incorporated into lipid cores. SLNs protect against the hydrolysis and oxidation of drugs and enhance bioavailability [174]. In a subsequent study, SLNs delivered RSV to skin keratinocytes in skin cancer. Fluorescence images demonstrated unequivocally that SLNs smaller than 180 nm migrate swiftly through cell membranes. They disperse throughout the cytoplasm, transit progressively along several cellular levels, and localize in the perinuclear region without cytotoxicity [175].

**Figure 10:**
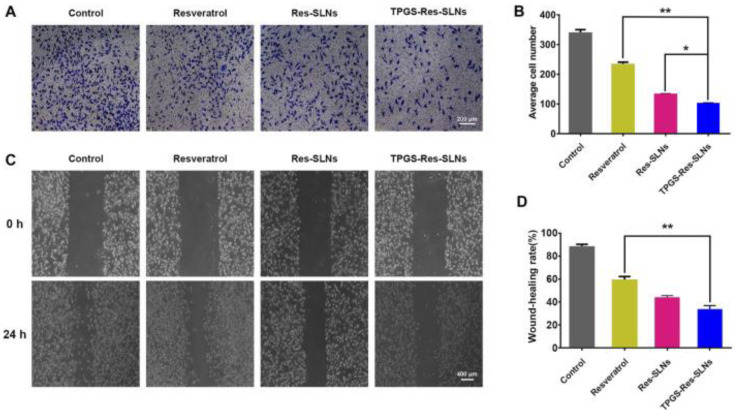
The invasion and migration ability of SKBR3/PR cells (**A**). Transwell migration assay was used to detect cell movement. Representative microphotographs of the Boyden chamber assay of SKBR3/PR cells. (**B**) The quantitative data for the Boyden chamber assay. The bar graph represents the number of invasive cells present per unit area in different treated groups. (**C**) Wound-healing assays were conducted to analyze cell migration. (**D**) Distance migrated by cells after 24 h. ** *p* < 0.01, * *p* < 0.05. Reprinted from Ref. [174]. Copyright 2021, *Frontiers in Bioengineering and Biotechnology*. This work is licensed under the Creative Commons Attribution License (CC BY).

**Figure 11:**
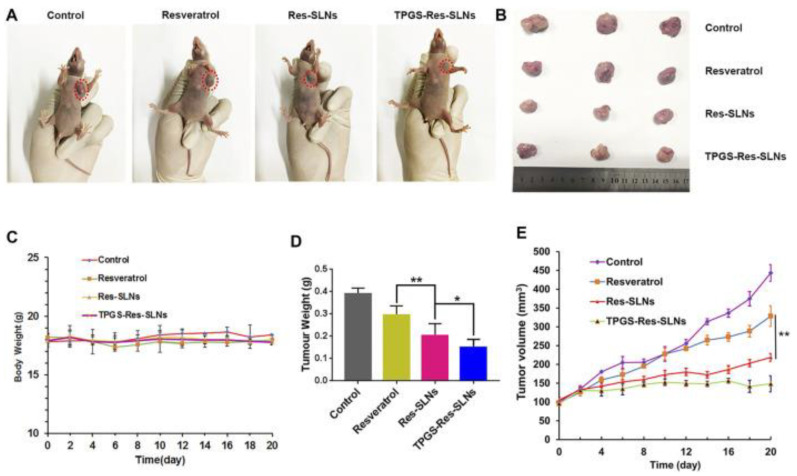
The anticancer effects of resveratrol and resveratrol loaded-SLNs on mice bearing SKBR3/PR xenografts. (**A**) Images of mice on the 16th day in different treatment groups. (**B**) Images of tumors excised from representative mice after the indicated treatments. (**C**) Body weight vs. time curves for mice treated with the indicated formulations. (**D**) Tumor weight of mice in the different treatment groups. (**E**) Tumor volume vs. time curves for mice treated with a variety of four formulations. * *p* < 0.05, ** *p* < 0.01. Reprinted from Ref. [174]. Copyright 2021, *Frontiers in Bioengineering and Biotechnology*. This work is licensed under the Creative Commons Attribution License (CC BY).

According to Wang et al., D-α-Tocopheryl polyethylene glycol 1000 succinate–resveratrol–solid lipid nanoparticles with resveratrol-loaded SLNs (TPGS-RSV-SLNs) induce cell death in SKBR3/PR cells and SKBR3/PR xenograft tumor models more efficiently than free RSV (Figure 10 and Figure 11) [174]. Song et al. designed resveratrol-loaded lipid–polymer hybrid nanoparticles (LPNs) and evaluated the anticancer activity in NSCLC, HCC827, NCIH2135, and HUVEC cell lines and BALB/c nude mice. The study results indicate that DTX/RSV LPNs have promising anticancer effects and low systemic toxicity [176].

In one study, the in vitro and in vivo efficacy of trans-resveratrol-loaded lipid-core nanocapsules (RSV-LNCs) against glioma cells was studied. RSVLNC reduced C6 glioma cell viability in vitro more than RSV alone. RSV-LNCs elicited early arrests at the S and G1 cell cycle stages, followed by apoptotic cell death, compared to RSV in solution; RSV-LNCs significantly reduced tumor size and certain malignant tumor-associated features in in vivo circumstances [177,178,179].

### 3.2. Inorganic Nanoparticles

#### 3.2.1. Gold Nanoparticles

Gold nanoparticles (GNPs) have been synthesized in a variety of shapes and structures, including nanospheres, nanorods, nanocubes, nanobranches, nanobipyramids, nanoflowers, nanoshells, nanowires, and nanocages. These particles are different from gold nanoparticles. GNPs are denser and are yellow inert solids with diameters ranging from 1 nm to 8 mm. Due to their novel optoelectronic and physicochemical features, gold nanoparticles (AuNPs) are increasingly used as components of medicinal solutions that target a variety of ailments, including cancer, neurological disease, and hepatitis. Recently, AuNPs have been physiologically synthesized using phytochemicals. Phytochemical nanoformulations offer improved cellular absorption, bioavailability, and anticancer action. Resveratrol-conjugated gold nanocomposites (RSV-GNCs) have a more substantial anticancer impact than RSV treatment alone.

Zhang et al. reported that RSV-GNCs exhibit a high anticancer effect on HepG2 cells compared to free RSV in terms of reducing cell proliferation, increasing apoptosis by upregulating caspase-8 and Bax, and downregulating pro-caspase-9, pro-caspase-3, PI3K, and Akt. In xenograft tests, RSV-GNCs significantly reduced vascular endothelial growth factor (VEGF) expression in tumor tissue, stimulated apoptosis, and inhibited tumor growth [180]. In one study, Park et al. evaluated the anticancer efficacy of RSV-GNCs on 12-O-tetradecanoylphorbol 13-acetate (TPA)-stimulated breast cancer cells (MCF-7). TPA increases invasion and migration activity in breast cancer cells. It was found that RSV-GNPs decreased the migration and invasion induced by TPA. RSV-GNPs markedly reduced NF-kB and AP-1 activation in TPA-stimulated breast cancer cells. Tumor development is highly linked to PI3K/Akt and MAPKs. PI3K/Akt and MAPK signaling also control invasion and metastasis-related molecules like MMPs and COX-2. Res-GNPs inhibit PI3K/Akt and MAPK: two biological processes involved in tumor development. Different malignancies are regulated in part by PI3K/Akt and ERK [181]. Lee et al. synthesized RSV-conjugated gold nanoparticles via polyvinylpyrrolidone (PVP) (cross-linked) (RSV-PVP-GNPs) and used Raw264.7 murine macrophage cells to evaluate their anticancer activity. Compared to free RSV, RSV-PVP-GNPs increased S-phase cell cycle arrest and apoptosis. RSV-PVP-GNPs trigger more aggressive apoptosis via intrinsic mitochondria compared to free RSV. In preclinical trials, RSV-PVP-GNPs conjugated with AS1411 aptamer effectively suppressed tumor volume without renal toxicity [182].

#### 3.2.2. Zinc Oxide Nanoparticles

Zinc oxide nanoparticles (ZnONPs) have been used for cancer diagnosis, drug delivery, and treatment [183]. ZnONPs are also used in the textile industry, cosmetics, and electronics [184]. Many methods are used to synthesize ZnONPs, such as physical, chemical, and biological practices [185]. Microemulsion, precipitation, hydrothermal procedures, and sol–gel are examples of chemical methods [186]. Ultrasonic irradiation, plasma, and vapor deposition are examples of physical methods [187]. All types of ZnoNPs have efficient anticancer compound release [188]. To utilize the particulate nature of ZnO NPs, RSV conjugated with ZnONPs (RSV-ZnONPs) has been developed to study the anticancer effect in cancer cell lines and animal models [43,189].

**Figure 12:**
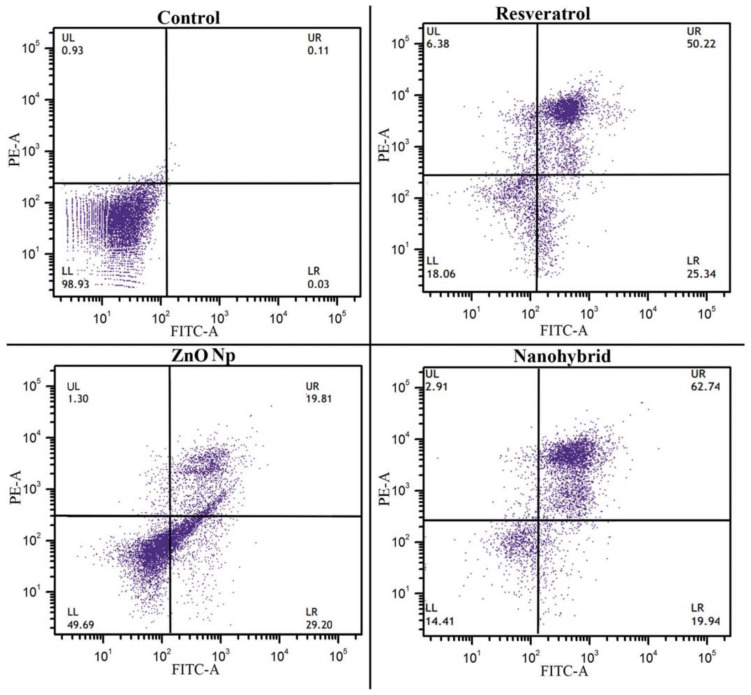
The apoptotic effect of RSV, ZnO, and RSV–ZnO on PA1 cells. Annexin V-FITC/PI-stained cell-representative dot plots of four independent experiments are presented. Reprinted with permission from [43]. Copyright 2016. The Royal Society of Chemistry.

**Figure 13:**
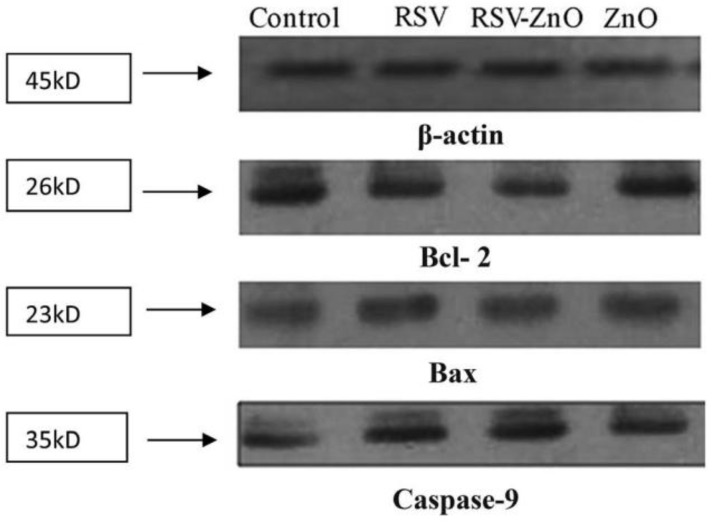
Western blot analysis of proteins. Bax, Bcl-2, and caspase-9 proteins in PA1 cells treated with RSV, RSV–ZnO and ZnO, with their IC50. Reprinted with permission from [43]. Copyright 2016. The Royal Society of Chemistry.

Khatun et al. designed RSV-ZnONPs to investigate their anticancer efficacy in ovarian cancer PA1 cell lines and animal models. The results showed that RSV-ZnONPs induced apoptosis in the PA1 cell line more effectively than free RSV. To confirm the apoptotic pathway of cell apoptosis, a Western blot assay was performed for the expression of caspase-9, Bax Bcl-2 in PA1 cells (Figure 12 and Figure 13) [43].

#### 3.2.3. Zeolitic Imidazolate Framework-8 Nanoparticles (ZIF-8 NPs)

Zeolitic imidazolate frameworks (ZIFs) belong to the subfamily of metal–organic frameworks (MOFs) [190]. Their topology is similar to that of zeolites [191]. Their structure comprises tetrahedral metal ions (e.g., Cu, Co, Zn, Fe) linked with four imidazolates [192]. The metal–imidazole–metal angle is expected to be the Si-O-Si angle in zeolites [193]. ZIFs possess the properties of both zeolites and MOFs, such as porosity, crystallinity, and chemical and thermal stability [194]. ZIFs are newly discovered materials that have attracted interest in various research fields. Around 13 ZIFs, including ZIF-4, ZIF-62, and ZIF-76, have been prepared in a glassy state [195]. ZIF-8 is constructed from zinc ions and 2-methylimidazolates through a coordination bond [196]. ZIF-8 has high encapsulation affinity and stability for therapeutic drugs compared to traditional drug delivery systems such as nanomicelles, polymeric nanoparticles, and liposomes [197]. ZIF-8 has high biodegradability, biocompatibility, and pH-responsive biodegradation properties [198]. ZIF-8 is disintegrated by the tumor’s acidic environment and releases therapeutic drugs. For this reason, ZIFs are used to carry therapeutic drugs in precision-targeted drug delivery systems [199].

**Figure 14:**
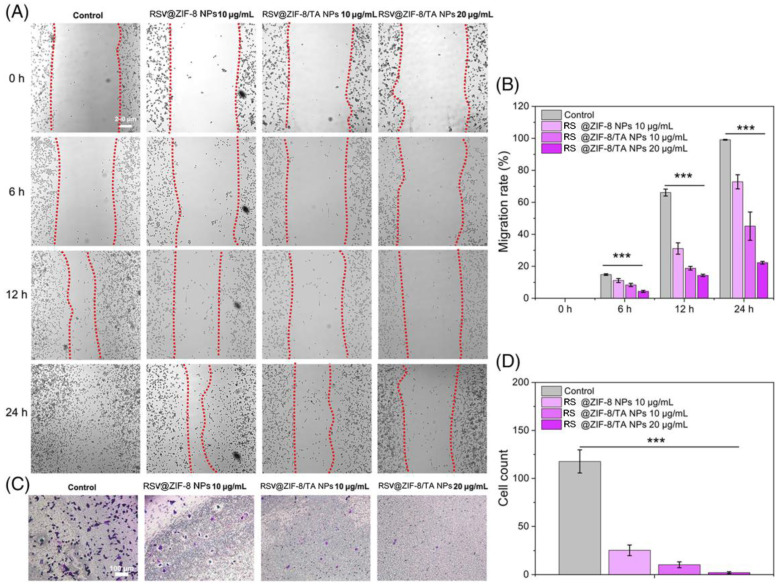
Cell scratch assay and Transwell assay. (**A**) Cell scratch assay with MC38 for 0, 6, 12, and 24 h. (**B**) Migration rate. (**C**) Transwell assay for 12 h. (**D**) Cell count. *** *p* < 0.001. Reprinted with permission from [200]. Copyright 2023. John Wileys and Sons.

**Figure 15:**
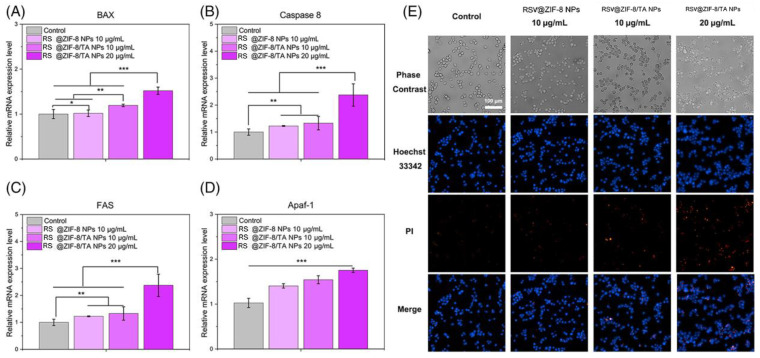
The apoptosis-related gene expression of MC38 cells. (**A**) BAX gene expression; (**B**) expression of caspase-8 gene; (**C**) expression of FAS gene; (**D**) expression of Apaf-1 gene; (**E**) Hoechst 33342 (blue)/PI (red) staining. * *p* < 0.05; ** *p* < 0.01; *** *p* < 0.001. Reprinted with permission from [200]. Copyright 2023. John Wileys and Sons.

Sun et al. synthesized RSV-loaded ZIF-8 nanoparticles using the “one-plot method”. The structure was modified with tannic acid (TA). TA provides a prolonged circulation time and increases biocompatibility. The anticancer properties of RSV-loaded ZIF-8 nanoparticles were evaluated against the MC38 cell line. The Transwell and cell scratch assay results showed that RSV-loaded ZIF-8 inhibits cancer cell invasion and migration. The Hoescht 33342/PI and RT-qPCR results demonstrated that RSV-loaded ZIF-8 nanoparticles upregulate apoptotic gene expression in cancer cells (Figure 14 and Figure 15) [200].

#### 3.2.4. Mesoporous Silica Nanoparticles

A mesoporous silica nanoparticle (MSN) is one of the different kinds of inorganic nanoparticles. It has gained significant attention due to its acceptable characteristics, such as size, shape, morphological features, porosity, surface area, physiochemical properties, high dispersion, and stability [201,202,203]. MSNs are constructed by the reaction of a template made of micellar rods with tetraethyl orthosilicate. After the reaction, the nano-sized rods are collected with a regular sequence of pores [202]. MSNs can also be produced using a simple sol–gel method called the spray-drying method, or the Stober process [204,205]. Around 1970, a substance that produces mesoporous silica was patented [206]. It almost remained unnoticed and was replicated in 1997 [207]. In 1990, scientists in Japan created mesoporous silica nanoparticles (MSNs) separately. They were subsequently produced in the laboratory of the Mobil Corporation and given the name Mobil Composition of Matter (or Mobil Crystalline Materials, MCM) [208].

Chaudhary et al. encapsulated MSNs with RSV and investigated their anticancer properties in PC3 prostate cancer cell lines and animal models. The results showed that synthesized nanoparticles with RSV had better antiproliferative activity than free RSV [209].

In another study, MSNs loaded with RSV (MSN-RSV) were constructed, and their anti-proliferation and cytotoxic properties in MGF-7 breast cancer cell line and BALB/c nude mice were evaluated. The results showed that MSN-RSV inhibits the NF-κB signaling pathway and has more cytotoxic and apoptotic effects than free RSV [210].

**Figure 16:**
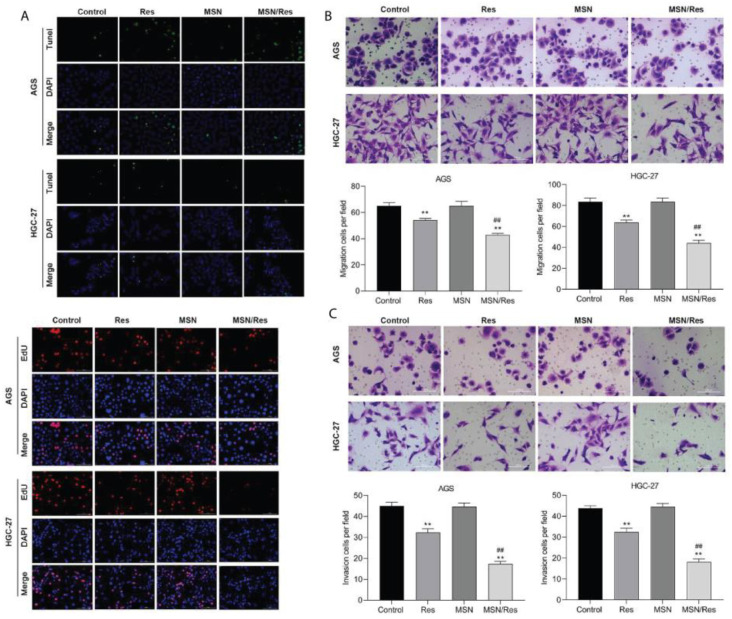
Proliferative and migration cellular assays in vitro and in vivo. (**A**) HGC-27 and AGS cell proliferation after treatment with Res, MSN-SH, or Res-loaded MSN was confirmed by EdU assay. (**B**) Apoptosis after treatment with Res, MSN-SH, or Res-loaded MSN was detected by terminal deoxynucleotidyl transferase biotin-dUTP nick end labeling (TUNEL). (**C**) Migration and invasion after Res, MSN-SH, or Res-loaded MSN treatment were detected by Transwell analysis. **, *p* < 0.01, Res group vs. MSN-SH or Control group; ##, *p* < 0.01, MSN/Res group vs. Res group. Reprinted from Ref. [211]. This work is licensed under Attribution-NonCommercial-No Derivatives 4.0 International (CC BY-NC-ND 4.0).

**Figure 17:**
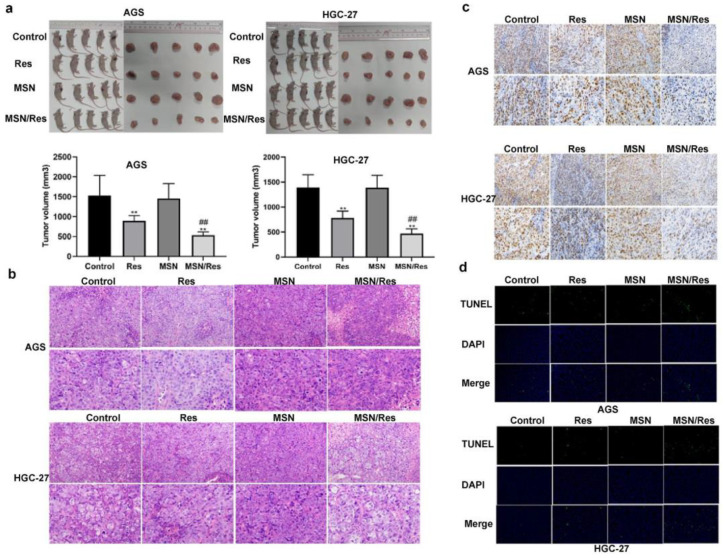
Biopsy and histology analysis of animal models. (**a**) Tumor size in HGC-27 and AGS tumor-bearing nude mouse models treated with Res, MSN-SH, or Res-loaded MSNs; (**b**) inflammatory cell infiltration (H&E staining). Magnification; (**c**) Ki67 immunohistochemical staining of HGC-27 and AGS cells in tumor-bearing nude mice; (**d**) apoptosis of HGC-27 and AGS cells treated with Res, MSH-SH, or Res-loaded MSNs, detected by TUNEL. **, *p* < 0.01, Res group vs. MSN-SH or Control group; ##, *p* < 0.01, MSN/Res group vs. Res group. Reprinted from Ref. [211]. This work is licensed under the Attribution-NonCommercial-No Derivatives 4.0 International (CC BY-NC-ND 4.0).

Lin et al. used the gastric cancer cell line HGC-27 and HGC-27-tumor-bearing mice to analyze resveratrol-loaded mesoporous silica nanoparticles’ (MSN-RSV) anticancer activity. The results indicated that, in in vitro and in vivo assays, MSN-RSV was more effective at reducing gastric cancer growth, invasion, and migration (Figure 16 and Figure 17) [211]. Summerlin et al. designed RSV-loaded MCM-48 (RSV-MCM-48) nanoparticles, whose anticancer efficacy was evaluated against colon cancer cell lines HT-29 and LS147T. They demonstrated that these nanoparticles have significant anticancer efficacy compared to free RSV, inhibiting the expression of the PARP and cIAP1 genes [212].

Marinheiro et al. explained that RSV-MSNs have excellent in vitro cytotoxicity on A375 and MNT-1 cell lines, decreasing cell viability [213].

#### 3.2.5. Carbon Nanotubes

Carbon nanotubes (CNTs) were discovered by Iijima and his coworkers in 1991. CNTs are carbon graphitic, hollow, and ordered nanostructures with a large surface area and light weight. CNTs’ diameter range is 1–100 nm. Both ends of the tubes are capped with half of the fullerene molecule. Tubes are cylinders of one or more different coaxial graphite layers. Every atom is linked to three neighbors, which provides structural strength [214]. CNTs are classified into two types based on their structure: single-walled carbon nanotubes (SWCNTs) and multi-walled carbon nanotubes (MWCNTs). SWCNTs have a diameter of 0.5 to 2.0 nanometers. They can be idealized as cutouts from graphene sheets rolled to form a hollow cylinder. MWCNTs are more complex than SWNTs with innumerate configurations of graphene building blocks. The structural arrangement involves a concentric arrangement of successive tubes increasing in diameter. Small tubes are contained within and turn into larger ones. MWNTs are composed of an unlimited number of walls [215]. CNTs are being used for diagnostic purposes, detecting proteins and DNA, identifying various types of proteins from serum, and delivering drugs. CNTs are used in target drug delivery systems for cancer therapies. CNTs cross various biological barriers, pass through the plasma membrane, and enter the cytoplasm, which helps deliver drugs to target sites. CNTS are considered important candidates for drug delivery due to their needle-like structure, biocompatibility, and high surface area [216]. A drug molecule can be delivered via CNTs in three ways: 1. drugs can be encapsulated in CNT cavities; 2. drugs can be conjugated using chemical tethers; 3. drugs can be linked to CNT amines or carboxylic acids [217].

**Figure 18:**
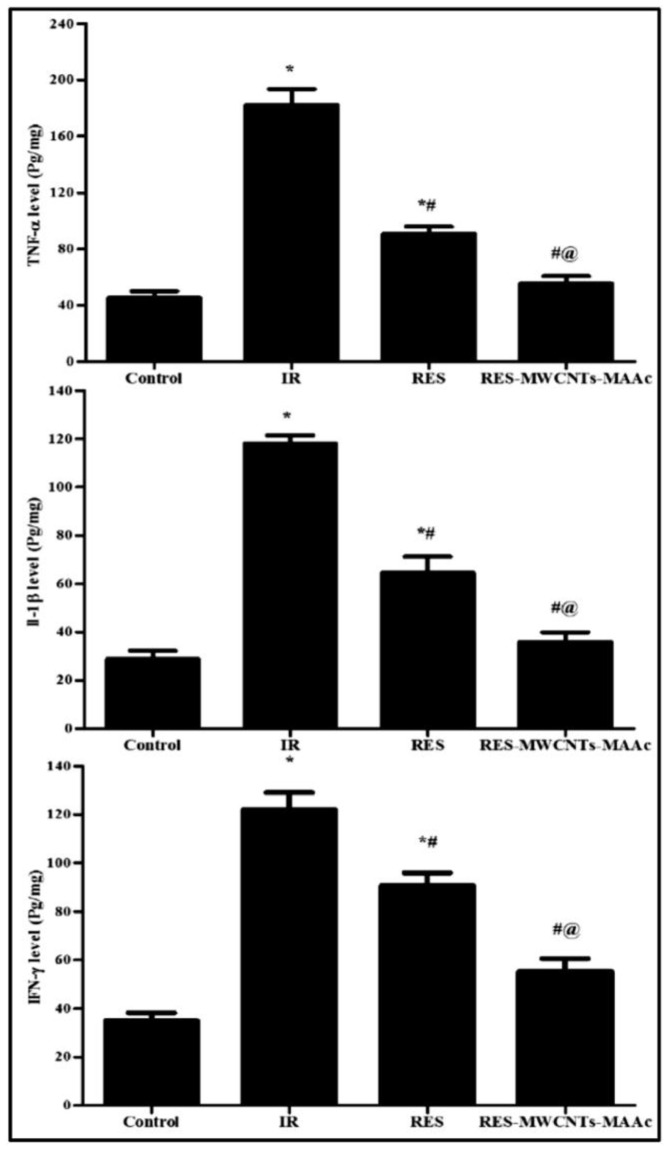
The expression of TNF-α, IL-1β, and IFN-γ in rats after oral administration of free resveratrol and resveratrol loaded-MWCNTs-MAAc. * significantly different from control group at *p* < 0.05, # significantly different from irradiated group at *p* < 0.05, @ significantly different from free RES group at *p* < 0.05. Reprinted with permission from Ref. [220]. Copyright 2021, Elsevier.

**Figure 19:**
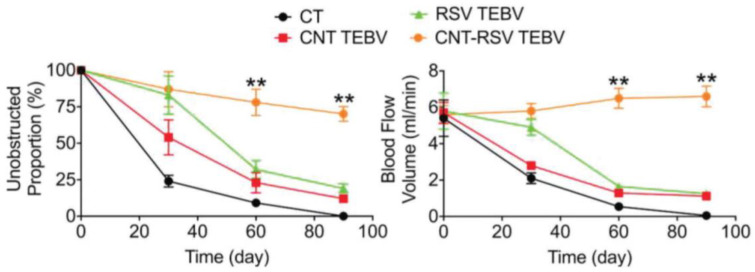
The quantification of unobstructed proportion of transplanted TEBVs and blood flow volume. Significant differences appeared on day 60. ** (*p* < 0.01). Reprinted with permission from Ref. [221]. Copyright 2018, John Wiley and Sons.

The surfaces of MWCNTs can be modified to enhance water solubility and increase the attachment site for drug encapsulation. For this purpose, hydrophilic functional groups can be linked with MWCNTs, or amphiphilic macromolecules such as polymers, surfactants, and lipids can be non-covalently linked with MWCNTs [218]. In one study, poly(acrylic acid) was covalently linked with the surface of MWCNTs through an atom transfer radical polymerization technique to increase bioavailability and enhance the controlled drug-release properties [219]. In another study, methacrylic acid (MAAc) was linked with MWCNTs and RSV (RSV-MWCNTs-MAAc) to evaluate its therapeutic potential against radiation-induced enteropathy in rats. In rats, the oral administration of RSV-MWCNTs-MAAc reduced the inflammatory mediators TNF-α, IFN-γ, and IL-1β. RSV-MWCNTs_MAAc showed more efficiency than free RSV due to prolonged RSV release at the tumor site (Figure 18) [220]. In another study, RSV was encapsulated in CNTs and conjugated with tissue-engineered blood vessels (TEBVs) (CNT-RSV TEBV), which resulted in the controlled release of RSV for up to 90 days compared to RSV TEBVs (Figure 19) [221].

#### 3.2.6. Bioactive Glass Nanoparticles

Bioactive glass (BG) is a glass–ceramic biomaterial made up of SiO_2_, Na_2_O, CaO, and P_2_O_5_. The discovery of bioactive glass was made by Larry Hench in 1969. Initially composed of 46.1% SiO_2_, 24.4% NaO, 26.9% CaO, and 2.6% P_2_O_5_ (in mol%), the pioneering bioactive glass was termed 45S5 bioglass [222]. Various methodologies have been used to synthesize bioactive glass nanoparticles (BGNs), such as sol–gel, melt-quenching, microwave irradiation, and flame synthesis [223]. It is reported that bioactive glass is used in bone tissue regeneration [224,225,226]. Recent studies have shown that the preparation of bioactive glass nanoparticles within the (SiO_2_-CaO-P_2_O_5_) system can be specifically tailored for osteomyelitis treatments [227,228,229]. It is also reported that BG has other applications, including cancer treatment strategies like hyperthermia, phototherapy, and anticancer drug delivery, in which BG has demonstrated efficacy [230,231,232]. Mesoporous bioactive glass (MBG) nanoparticles have been used for high drug load and targeted therapy approaches. Surface modification techniques, such as functionalization and composite production involving polymers and hydrogels, have been employed to enhance drug-release kinetics [233]. In one study, gold nanoparticles were incorporated into a BG–chitosan–gelatin composite, resulting in a significantly higher loading for the anticancer drug doxorubicin (DOX) compared to magnetic-core silica nanoparticles [234,235]. The modification of bioactive glass surfaces with folic acid has been used to exploit the molecular targeting of tumor cells that overexpress folic acid receptors. This strategy facilitates the internalization of the glass by cancer cells, thereby enhancing the anti-tumor properties of drug [236].

Cazzola et al. found polyphenol-grafted bioactive glass showed selective cytotoxic activity against human bone osteosarcoma (U2OS) cells when cultivated directly onto the glass surface. Furthermore, the presence of grafted polyphenols increased the production of reactive oxygen and nitrogen species (RONS), inducing permanent DNA damage in U2SOS cells while displaying anti-inflammatory effects on human fetal pre-osteoblasts (hFOB). These findings suggest that polyphenol-grafted bioactive glass holds promise as a material for bone substitution in cancer treatment [237].

Another study conducted by Dziadek et al. involved the synthesis of bioactive glass composites with polyphenols from sage using the solvent-casting method. These compositions exhibited significant antiproliferative and antioxidant effects against the WM266-4 cell line [238]. In another study, a bioactive glass composite with resveratrol (RSV) and chitosan demonstrated a dose-dependent decrease in the expression of cytokines TNF-α, IL-1β, and iNOS when tested against the RAW264.7 cell line [239].

The nanocomposite of bioactive glass and resveratrol for anticancer treatment represents an understudied domain, both in vitro and in vivo. The current research in this field is limited, creating significant knowledge gaps concerning the optimal formulation, cellular interactions, and therapeutic outcomes associated with bioactive glass nanoparticle-conjugated resveratrol. The limited available studies highlight a promising area for further investigation and potential breakthroughs in cancer therapeutics. The absence of detailed research at this specific intersection urges the need for dedicated efforts to explore the effects of bioactive glass nanoparticles and resveratrol in the context of anticancer therapies. The opportunity to fill these knowledge gaps can pave the way for innovative approaches and relevant discoveries in the treatment of cancer.

**Table 1:** Summary of preparation techniques, drug-release mechanisms, and pros and cons of different nanoparticles.

Types of Nanoparticles	Preparation Techniques	Drug-Release Mechanism	Pros	Cons	Ref.
Liposomes	Reverse-phase evaporation–dehydration–rehydration, solvent injection, and microfluidic-based techniques.	Diffusion-controlled. Triggered release.	Encapsulation of hydrophilic and hydrophobic drugs. Biocompatibility.	Potential for drug leakage.	[127]
Solid lipid nanoparticles	High-pressure homogenization, emulsification, high-speed stirring, and ultrasonication method.	Diffusion-controlled. Matrix erosion.	Sustained release and stability.	Limited drug compatibility. Potential for aggregation.	[175]
Dendrimers	Divergent, convergent synthesis.	Diffusion-controlled. Surface-charge-driven.	Controlled release. High drug payload.	Complex synthesis. Immunogenicity.	[168]
Polymeric nanoparticles	Solvent diffusion, solvent evaporation, ionic gelation, self-assembly, polymer electrostatic interaction, desolvation, and emulsion techniques.	Diffusion-controlled. Chemically controlled.	Sustained release. Potential for targeted delivery.	Batch-to-batch variability. Potential toxicity.	[145]
Gold nanoparticles	Chemical reduction, citrate reduction, seed-mediated.	Temperature-dependent release. Controlled release, pH-dependent release.	High stability, tunable size and shape. Excellent biocompatibility.	Potential toxicity, limited biodegradability. Limited drug compatibility, potential aggregation. Potential impact on the immune system.	[240]
Zinc oxide nanoparticles	Sol–gel method, precipitation, hydrothermal synthesis	pH-dependent. Temperature-dependent release.	Potential for targeted drug delivery. UV protection for various applications.	Toxicity and biocompatibility concerns. Ecotoxicity concerns.	[241]
ZIF-8 nanoparticles	Solvothermal, microwave-assisted, co-precipitation.	pH-dependent release. Guest molecule exchange.	High porosity, tunable size, and structure. Biocompatible carrier.	Limited biodegradability. Potential toxicity. High power usage.	[242]
Mesoporous silica nanoparticles	Sol–gel, co-condensation method.	pH-dependent release. Stimulus-responsive systems. Controlled guest molecule release.	Excellent biocompatibility. High pore size. Biocompatible carrier. Enhanced drug stability in the pores.	Limited biodegradability. Potential toxicity. Aggregation and pore collapse.	[243]
Carbon nanotubes	Chemical vapor deposition, arc discharge, laser ablation method.	pH-dependent release. Temperature-sensitive release.	Large surface area. Highly efficient drug delivery. Lightweight. Biocompatible.	It may induce inflammation and immune response.	[244,245]
Bioactive glass nanoparticles	Sol–gel synthesis, flame synthesis, precipitation methods.	Ion exchange between the glass matrix and the surrounding environment.	Osteoinductive properties. Biocompatibility.	Limited loaded capacity. High cost.	[233]

**Table 2:** Summary of antioxidative, antiproliferative, and anticancer activities of resveratrol-loaded organic nanoparticles.

Drug	Organic Nanoparticle Formulation	Target System	Major Findings	Ref.
RSV	Polymeric micelles	PC12 cell lines	Protect cells against Aβ-induced damage by attenuating oxidative stress and affecting apoptosis without long-term cytotoxicity.	[163]
RSV	Solid lipid nanoparticles (SLN)	NCTC2544 cell lines	Decreases cell proliferation. High cytostatic effect of SLN–RSV in contrast to free RSV.	[175]
RSV + QUE	Liposomes	HDFa cell lines and CD-1 mice	It increases apoptosis and decreases leukocyte infiltration.	[130]
RSV	Lipid-core nanocapsules	C6 glioma cell lines and rats implanted with C6 glioma cells	Tumor size decreases compared to free RSV.	[179]
	Transferrin-modified PEGylated liposomes	Xenograft mouse model of GBM and U-87 MG cell lines	Induce a high level of apoptosis and cytotoxicity compared to free RSV. Tumor growth inhibition and increased survival rate in mice.	[131]
	Transferrin (Tf) modified poly ethylene glycol-poly lactic acid (PEG-PLA) nanoparticles	C6, U87 cell lines and brain-glioma-bearing rat model	Cytotoxicity in C6 and U87 cells was higher than that of free RSV. Tumor volume decreases compared to free RSV.	[159]
	Folate-modified nanostructured lipid carriers	MCF-7 cell lines and female Wistar rats	High antitumor effect of folate-modified NLCs (RSV-FA-NLCs).	[138]
PTX and RSV	PEGylated liposome	MCF-7 cell lines and BALB/c nude mice	Increase the bioavailability of the drugs in vivo. Liposome exhibits potent cytotoxicity against the drug-resistant MCF-7/ADR tumor cells. Liposomes improve the bioavailability of the drugs and enhance drug retention in the tumor.	[133]
EXM/RSV	Zein nano-capsules	MCF-7, 4T1 cell lines, and female Sprague Dawley rats	Increased antitumor activity in cell lines. Reduced tumor volume in mice by 2.4-fold compared to free RSV.	[162]
RSV	Solid lipid nanoparticles	SKBR3/PR, SKBR3/PR xenograft tumor models	More apoptosis of cancer cells. Inhibit cell migration compared with free RSV.	[136]
RSV	Dequalinium polyethylene glycol-distearoyl Phosphatidyl ethanolamine	Xenografted resistant A549/cDDP nude mice	Cellular uptake is enhanced with induced apoptosis of non-resistant and resistant cancer cells.	[132]
RSV + CUR	Liposomes	PTEN-CaP8 cancer cell lines and B6C3F1/J mice	Inhibit cell growth and induce apoptosis. Decrease prostatic adenocarcinoma in vivo.	[96]
RSV	Poly(epsilon-caprolactone) (PCL) and poly (d,l-lactic-co-glycolic acid)-poly(ethylene glycol) conjugate (PLGA-PEG-COOH)	DU-145, PC-3, and LNCaP cell lines	Increased cytotoxicity compared to that of free RSV.	[124]
DOX and RSV	PLGA nanoparticle	BALB/c nude mice and MCF-7/ADR and MDA-MB-231/ADR cell lines	Increase cytotoxicity in vitro. Inhibit the DOX-resistant tumor growth in vivo without causing systemic toxicity.	[151]
RSV	PLGA-polyethylene glycol (PEG) NPs coated with chitosan	Athymic mice	Increases bioavailability and reduces tumor growth compared to free RSV.	[152]
RSV	Biomimetic nanocarrier	HT29 and HCT116 cell lines and C57/BL6j female nude mice	Extended circulation effect. A significant antitumor efficacy was observed in vivo.	[154]
RSV	Epidermal growth factor (EGF) conjugated lipid–polymer hybrid nanoparticles	HCC827, NCIH2135, and HUVEC cell lines and BALB/c nude mice	High tumor inhibition and less organ toxicity.	[176]

**Table 3:** Summary of anticancer activities of resveratrol-loaded inorganic nanoparticles.

Drug	Inorganic Nanoparticle Formulation	Target System	Major Findings	Reference
RSV	Gold nanoparticles	HepG2 cells	Reduce cell proliferation and increase apoptosis by upregulating caspase-8 and Bax, and downregulating pro-caspase-9, pro-caspase-3, PI3K, and Akt.	[180]
RSV	Gold nanoparticles	RAW264.7	RSV-GNPs increased S-phase cell cycle arrest and apoptosis compared to free RSV.	[182]
RSV	ZnO nanoparticles	PA1 cell lines and animal models	ZnO-NPs induce apoptosis more effectively in the PA1 cell line compared to free RSV.	[43]
RSV	Zeolitic imidazolate framework-8 nanoparticles	MC38 cell line	RSV-loaded ZIF-8 nanoparticles upregulate the expression of apoptotic genes in cancer cells.	[200]
RSV	Mesoporous silica nanoparticles	PC3 prostate cancer cell line	They have better antiproliferative activity than free RSV.	[209]
		MGF-7 breast cancer cell line and in BALB/c nude mice	MSN-RSV inhibit NF-κB signaling pathway and have a more cytotoxic and apoptotic effect than free RSV.	[210]
		Gastric cancer cell line HGC-27 and HGC-27-tumor-bearing mice	They are more effective at reducing the growth, invasion, and migration of gastric cancer both in vitro and in vivo.	[211]
		Colon cancer cell lines HT-29 and LS147T	They inhibit the expression of PARP and cIAP1 genes and show better anticancer efficacy than free RSV.	[212]
		A375 and MNT-1 cell lines	Excellent in vitro cytotoxicity, decrease the cell viability.	[213]
RSV	Carbon nanotubes	Wistar rats	Oral administration of methacrylic acid (MAAc) linked with multi-walled carbon nanotubes (MWCNTs) and RSV (RSV-MWCNTs-MAAz) reduced inflammatory mediators TNF-α, IFN-γ, and IL-1β RSV-MWCNTs-MAAc showed more efficiency than free RSV due to prolonged RSV release at the tumor site.	[220]

## 4. Conclusions and Future Perspectives

Resveratrol has gained much attention due to its role in reducing cancer risk and its function as a chemopreventive or cancer therapeutic agent. It inhibits various pathways, such as metastasis, angiogenesis, apoptosis, and autophagic cell death. However, many challenges hinder RSV development as a cancer treatment. These challenges include issues related to low bioavailability, rapid metabolism, drug interactions, and cytotoxicity. Researchers have developed different kinds of nanoparticles for RSV nanoformulation to overcome these obstacles, modifying different structural parameters.

**Figure 20:**
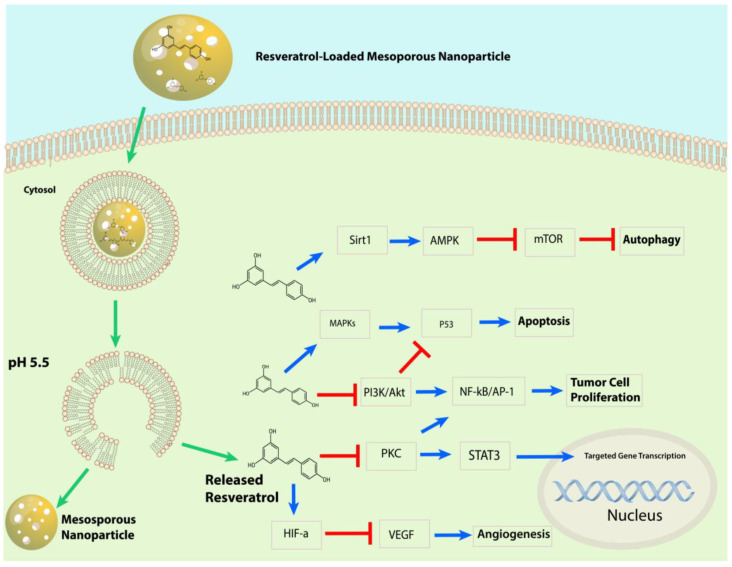
Summary figure depicting resveratrol-loaded nanoparticles and different possible anticancer mechanisms.

This review has highlighted the use and biological effects of nanoparticles for RSV delivery, as well as the primary state-of-the-art knowledge on improving properties like bioavailability, solubility, targeted drug delivery, efficiency, the induction of cancer cell death, and tumor reduction in animal models. Figure 20 shows the resveratrol-loaded nanoparticles and several possible anticancer mechanisms discussed in the manuscript.

Despite further research being needed to comprehensively assess the cytotoxicity, stability, optimal dosing, biocompatibility, and safety of nanoformulated RSV across various cancer types, preclinical and clinical trials will continue to be performed to understand nanoformulated RSV’s operational dynamics mechanisms in a translational way. Therefore, current preclinical trials of resveratrol-loaded nanoparticles (RSV-NPs) show promise, and the ongoing transition to clinical trials is a critical step in realizing their potential for cancer prevention and treatment.

While preclinical trials have shown significant promise, there are limitations and challenges that should be addressed in clinical translation. One challenge is the long-term toxicity profile; a comprehensive study is needed to ensure prolonged safe use. Moreover, determining the optimal dose is another challenge which requires a balance between therapeutic efficacy and minimizing adverse effects. Additionally, addressing pharmacokinetic details is essential for optimizing drug delivery and enhancing clinical outcomes. Furthermore, maintaining therapeutic efficacy while maintaining consistent and predictable manufacturing at an industrial scale is a complex challenge. It is foreseen that research on RSV-NPs in cancer treatment will continue to evolve with ongoing developments. While nanotechnology holds promise for improving RSV cancer therapeutics, persistent efforts are required to facilitate clinical RSV-NP translation.

The use of RSV-NPs holds significant promise for future research and clinical translation into cancer therapy. Anticancer therapies are expected to be increasingly personalized, with RSV-NPs tailored to patients’ genetic and molecular profiles, enhancing treatment outcomes. Additionally, exploring combination therapies of RSV-NPs with other therapeutic agents could lead to more effective cancer treatment strategies. Further, comprehensive biodistribution studies and long-term safety assessments are essential for ensuring RSV-NPs’ clinical use. With the integration of pharmacometrics and artificial intelligence systems applied to optical imaging (based on machine learning), the future offers exciting possibilities for improving the efficacy and safety of cancer therapies by encapsulating RSV and conveying it via ad hoc nanoparticles.

These perspectives provide a path forward for further investigations, with the clinical application of RSV-NPs as a possible cancer treatment approach.

In view of this, it is necessary to continuously update the state of knowledge on the various types and applications of nanoformulations suitable for drug administration, and therefore, regarding their biological effects on in vitro and in vivo samples, to implement and constantly enrich the drug discovery and development landscape.

## Data Availability

Data sharing is not applicable.
